# Racial and ethnic differences in fatal child abuse and neglect and the intersection of community poverty: U.S., 2003 to 2022

**DOI:** 10.1016/j.chipro.2025.100108

**Published:** 2025-04

**Authors:** Rebecca F. Wilson, Xin Yue, Karen E. Thomas, Krishna Kiran Kota, Carter J. Betz

**Affiliations:** a Division of Violence Prevention, National Center for Injury Prevention and Control, Centers for Disease Control and Prevention, Atlanta, GA, USA; b Division of Injury Prevention, National Center for Injury Prevention and Control, Centers for Disease Control and Prevention, Atlanta, GA, USA; c Division of HIV Prevention, National Center for HIV, Viral Hepatitis, STD, and TB Prevention, USA

**Keywords:** Fatal child abuse and neglect, Child abuse, Community poverty, Poverty

## Abstract

**Introduction:**

In the U.S., child abuse and neglect (CAN) is a significant public health problem. Poverty is a well-known correlate of CAN.

**Objective:**

Examine racial and ethnic differences in fatal CAN among U.S. children and the intersection of community poverty.

**Participants and methods:**

This study integrated National Violent Death Reporting System (NVDRS) data, county poverty data, and population estimates data. We used NVDRS data to examine fatal CAN for children aged 0–17 years for 2003–2022. Fatal CAN was defined as a homicide precipitated by abuse or neglect by a parent or caregiver. Racial and ethnic differences in fatal CAN were examined using pairwise comparisons. Community poverty quartiles for fatal CAN cases were determined using county-level poverty data and population estimate data for 2003–2022.

**Results:**

During 2003–2022, NVDRS captured 6182 fatal CAN cases; 57.3% were boys; 79.6% were aged 0–5 years. An argument (21.4%), child’s history of abuse (20.1%), and intimate partner violence (IPV; 15.6%) were the three most common precipitators of fatal CAN. IPV as a precipitator was most common among Asian or Pacific Islander (API; 33.0%), Hispanic (16.4%), and White (19.1%) victims than Black victims (10.8%; p < 0.05). More than one in ten (13.9%) fatal CAN deaths co-occurred with the perpetrator’s suicide; this occurred most commonly among API victims (38.1%; p < 0.05) than Black (5.8%), multiracial (13.4%), and White (13.9%) victims. A larger proportion of fatal CAN among API victims (14.2%; p < 0.05) was precipitated by a crisis than did fatal CAN of Black (3.3%), multiracial (4.7%), and White (4.5%) victims.

During 2003–2022, more than one in three (35.9%) fatal CAN victims resided in communities classified as the most impoverished; 52.7% of AI/AN victims resided in these communities, followed by Black (46.7%), Hispanic (31.3%), multiracial (30.9%), White (28.7%), and API (12.4%) victims. During this same period, 47.8% of API fatal CAN victims resided in communities with the least poverty, followed by White (17.3%), Hispanic (15.3%), multiracial (16.6%), and Black (10.1%) victims.

**Conclusions:**

Fatal CAN is preventable. Employing multiple strategies, at various levels (e.g., individual, familial, community), might aid in preventing nonfatal and fatal CAN.

## 1. Introduction

In the United States (U.S.), child abuse and neglect (CAN) is a significant public health problem (Leeb, Paulozzi, Melanson, Simon, & Arias, 2008). National CAN data indicates roughly 558,899 children were reported and confirmed to have experienced CAN in 2022; an estimated 1990 died from CAN (U.S. Department of Health & Human Services [USDHHS], The Administration for Children and Families [ACF], 2024a). Black or African American (Black), non-Hispanic (NH) children have the highest CAN fatality rate (6.4 per 100,000 children) in the U.S., followed by children of two or more races (multiracial; 4.0 per 100,000 children), American Indian or Alaska Native (AI/AN), NH children (3.4 per 100,000 children), White, NH children (2.0 per 100, 000 children), and Hispanic or Latino children (Hispanic; 1.7 per 100, 000 children) (USDHHS, ACF, 2024a).

Although fatal CAN involves a set of behaviors (e.g., physical assault, neglect) by a parent or caregiver (Leeb et al., 2008), research shows that a complex interplay between factors can either protect against or increase risk of CAN perpetration and victimization (Drake, Jonson-Reid, & Dvalishvili, 2022; Maguire-Jack & Font, 2017; USDHHS***, ACF, 2023/2024)***. Risk factors exist at various levels such as the individual-(parent or caregiver [e.g., maternal depression]), family-, or community-level (e.g., poverty); children exposed to a multitude of these factors are at an increased risk of experiencing CAN (Drake et al., 2022; Dubowitz et al., 2011; Maguire-Jack & Font, 2017).

Community poverty is a well-documented factor associated with fatal and nonfatal CAN (Drake et al., 2022; Farrell et al., 2017; Kim & Drake, 2023; Maguire-Jack & Font, 2017). Studies show families residing in communities with high poverty levels often experience heightened stress and material hardships (e.g., inadequate resources for housing) (Drake et al., 2022; Pelton, 1994), creating conditions that make it harder for parents and caregivers to nurture and protect their children (USDHHS***, ACF, 2023/2024)***. Understanding the intersection of fatal CAN and community poverty is important for prevention. The purpose of this study is to examine racial and ethnic differences in fatal CAN among U.S. children and how community poverty intersects with these deaths.

## 2. Methods

### 2.1. Study design and data

This cross-sectional study integrated three data sources: Centers for Disease Control and Prevention’s (CDC) National Violent Death Reporting System (NVDRS) (Nguyen et al., 2024), U.S. Census Bureau County poverty data, (U.S. Census Bureau, 2022), and U.S. Census Bureau Population Estimates data(U.S. Census Bureau, 2024). NVDRS data for child homicides, which includes fatal CAN cases, come from 52 states/jurisdictions for years 2003–2022. See Supplementary Table 1 for a list of states/jurisdictions included in this study and years and geographic coverage area for each. NVDRS is a state- and jurisdiction-based surveillance system that collects information on violent deaths, linking information from death certificates, coroner or medical examiner records, and law enforcement reports into one incident. Trained abstractors enter information into a web-based system using standardized coding guidance from the CDC. Additional details on NVDRS methodology are available elsewhere (Nguyen et al., 2024).

### 2.2. Case definition

Child homicides, which include fatal CAN, were defined using the World Health Organization’s International Classification of Diseases, 10th Revision, underlying cause of death codes X85-Y09 or Y87.1 for children aged 0–17 years. Fatal CAN is defined in NVDRS as a death precipitated by abuse (e.g., physical assault) or neglect (e.g., inadequate supervision) by a parent or caregiver (Centers for Disease Control and Prevention, 2022).

### 2.3. Fatal child abuse and neglect case identification

The following procedures were used to identify fatal CAN cases: 1) Since NVDRS did not begin systematically collecting fatal CAN data until 2013, NVDRS’s standard ‘suspect was a caregiver’ variable was used as a proxy to identify fatal CAN cases for child homicide victims who died in 2003–2012, yielding n = 1095 cases; 2) NVDRS’s standard ‘abuse or neglect led to death’ variable was used to identify fatal CAN cases from 2013 (the year this variable was added as an NVDRS circumstance) through 2022 (the most recent data available), yielding an additional n = 3729 cases; and 3) NonCAN-related homicides were reviewed if: a) ‘suspect was a caregiver’ variable was endorsed as *no* by the data abstractor, but intentional neglect or shaken baby syndrome was the method used in child homicide, or b) if ‘suspect was a caregiver’ variable was endorsed as *yes* by the data abstractor for data years 2013–2022, but ‘abuse or neglect led to death’ circumstance was endorsed as *no*, or c) if the perpetrator was a parent or caregiver (e.g., babysitter); this yielded an additional n = 1400 cases. Forty-two fatal CAN cases were excluded because child’s race and ethnicity were other, unspecified, unknown, or missing, yielding a total of 6182 cases for this study.

### 2.4. Statistical analyses

We examined differences in fatal CAN by child’s race and ethnicity using chi-square and Fisher’s exact tests, with posthoc non-parametric pairwise comparisons used to test significant results. Race and ethnicity categories used were AI/AN, non-Hispanic (NH) (referred to as AI/AN herein); Asian or Pacific Islander (API), NH (referred to as API herein); Black, NH (referred to as Black herein); Hispanic; White, NH (referred to as White herein); and multiracial, NH (referred to as multiracial herein). We examined race and ethnicity as indicators, not drivers, of inequities because race is a social construct and not a biological driver for disparities (Braveman & Dominguez, 2021). Two-sided p-values <0.05 were considered statistically significant. Analyses were performed using SAS Version 9.4 (SAS Institute Inc.).

### 2.5. Community poverty analysis

**Fig. 1.**
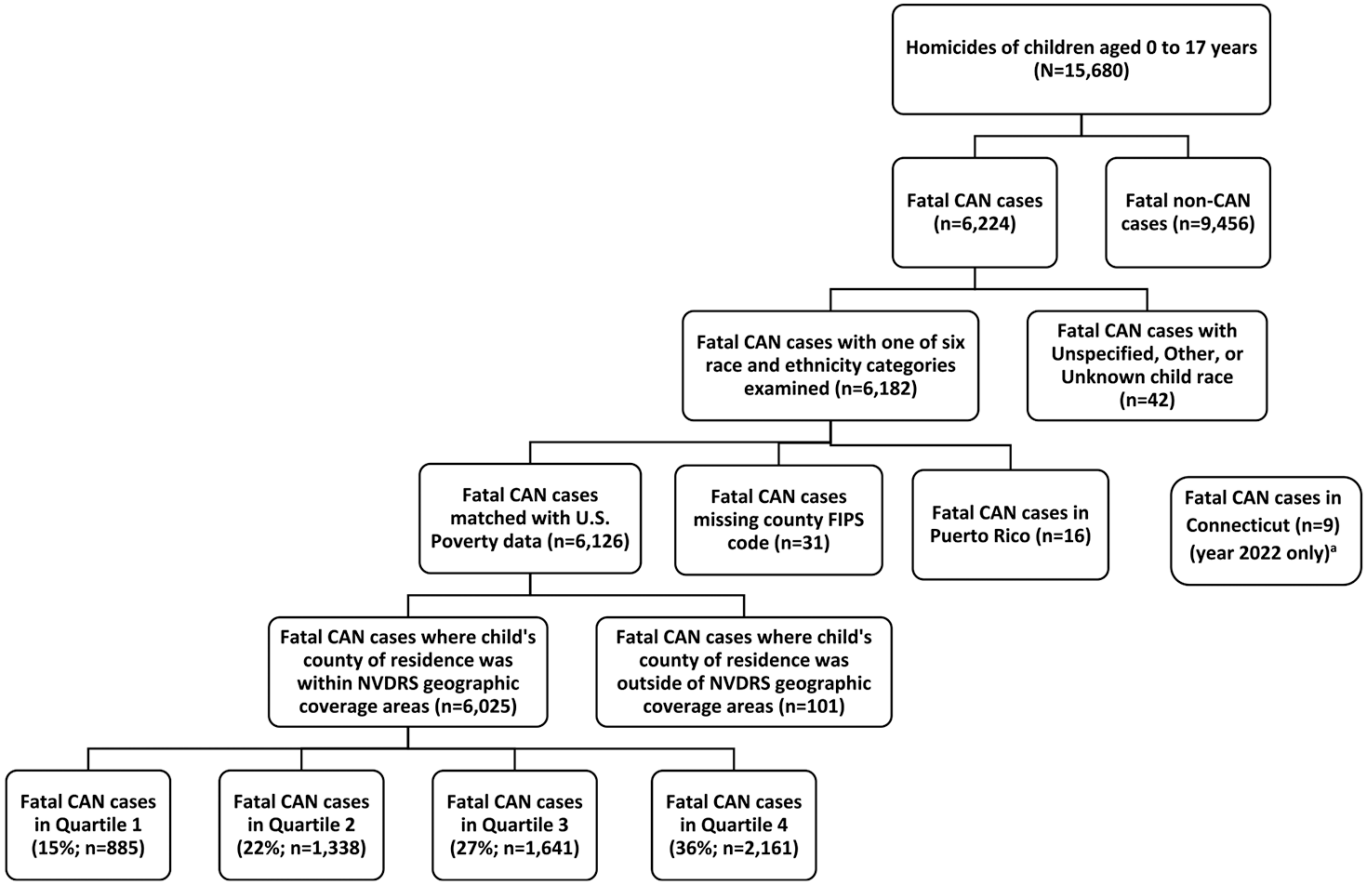
Flowchart of Fatal Abuse and Neglect Case Identification and Selection of Residing in Community Poverty among children aged 0–17 years, NVDRS, United States, 2003 to 2022 Abbreviations: CAN (Child Abuse and Neglect); Federal Information Processing System (FIPS); NVDRS (National Violent Death Reporting System) ^a^In 2022, approved by the U.S. Census Bureau, Connecticut adopted nine planning regions as county-equivalent geographic units, replacing data collected at the county-level. This change prohibited us from including Connecticut’s 2022 fatal CAN cases in the community poverty analysis.

Community poverty quartiles: lowest poverty level (Q1) through highest poverty level (Q4) for fatal CAN cases were determined using county poverty data (U.S. Census Bureau, 2022) and U.S. Population Estimate data for 2003–2022 (U.S. Census Bureau, 2024). See Supplementary Table 2. Poverty quartile ranges were determined for single years (See Supplementary Table 3). Counties participating in NVDRS programs (hereafter called NVDRS geographic coverage areas) were identified (See Supplementary Table 1), and poverty quartiles were generated so that approximately 25% of the population within NVDRS geographic coverage areas resided in each quartile for each year. Fatal CAN cases were then assigned to a poverty quartile based on the Federal Information Processing System (FIPS) code of the child victim’s county of residence. One hundred and fifty-seven fatal CAN cases were excluded from the community poverty analysis for the following reasons: 1) county FIPS code for child victim was missing, 2) data were from Puerto Rico because poverty estimates are unavailable for Puerto Rico, or 3) child victim’s county of residence was not within NVDRS geographic coverage areas (Fig. 1). Community poverty data for fatal CAN cases in quartiles one and four are presented in multiple years (i.e., 2003–2007; 2008–2012; 2013–2017; and 2018–2022) to account for small counts in some racial and ethnic groups for individual years. A total of 6025 fatal CAN cases (Q1: n = 885, Q2: n = 1,338, Q3: n = 1,641, Q4: n = 2161) were included in the community poverty analysis.

## 3. Results

### 3.1. Descriptive statistics

From 2003 to 2022, 15,680 homicides of children aged 0–17 years were included in NVDRS, of which 39.4% (n = 6182), resulting from CAN, were included in this analysis (Table 1). AI/AN and API children were fatal CAN victims in similar proportions at 1.9% (n = 119) and 1.8% (n = 113), respectively. Black, Hispanic, and White children accounted for 36.3% (n = 2246), 14.5% (n = 894), and 41.1% (n = 2541) of fatal CAN victims, respectively. Multiracial children accounted for 4.4% (n = 269) of fatal CAN victims.

#### 3.1.1. Victim characteristics

Fatal CAN victims were disproportionately boys (57.3%; n = 3539), infants (i.e., children aged <1 year; 35.7%; n = 2207), and those aged 1–5 years (43.9%; n = 2715). The largest proportion of fatal CAN victims were aged 1–5 years; this proportion was higher among AI/AN (52.9%; p < 0.05) and Black victims (48.0%; p < 0.05). See Table 1 for full results.

#### 3.1.2. Weapon type

When weapon type was known, personal weapons (e.g., hands, fists, feet; 23.1%; n = 1426), blunt instruments (16.9%; n = 1042), and firearms (15.2%; n = 937) were the three most common weapons used in fatal CAN. A larger proportion of Black fatal CAN victims (20.9%) were killed with blunt instruments than were Hispanic (15.2%; p < 0.05) and White victims (14.2%; p < 0.05). A larger proportion of AI/AN (33.6%), Black (24.4%), and Hispanic (25.5%) fatal CAN victims were killed with personal weapons than were White victims (21.2%; p < 0.05). A larger proportion of API (28.3%) and White (19.1%) fatal CAN victims were killed with firearms than were Black (11.2%; p < 0.05) and Hispanic victims (13.2%; p < 0.05). See Table 1 for full results.

#### 3.1.3. Perpetrator characteristics

When perpetrator sex was known, 59.3% of fatal CAN victims were killed by a male perpetrator. A larger proportion of API (47.2%), Hispanic (38.9%), multiracial (43.3%), and White (41.7%) fatal CAN victims were killed by their father than were Black victims (31.6%; p < 0.05). A larger proportion of Black fatal CAN victims (28.1%) were killed by their mother than were White victims (24.5%; p < 0.05).

#### 3.1.4. Precipitating circumstances

When circumstances were known, argument (21.4%) or child’s history of abuse (20.1%) were the two most common precipitators or characteristics in fatal CAN. Among fatal CAN victims, 15.6% involved intimate partner violence (IPV), meaning the homicide was connected to conflict between current or former intimate partners, but the child victims were not the intimate partner themselves; this precipitator was most common among API (33.0%), Hispanic (16.4%), and White (19.1%) victims than Black victims (10.8%; p < 0.05). Family relationship problems precipitated almost one in ten (9.9%) fatal CAN cases; this circumstance occurred most commonly among API (16.5%), Hispanic (15.5%), and White (10.5%) victims than Black victims (7.2%). More than thirteen percent (13.9%) of fatal CAN incidents co-occurred with suicide of the perpetrator; this occurred most commonly among API victims (38.1%; p < 0.05). A recent or impending crisis during the previous or upcoming two weeks precipitated 4.5% of fatal CAN; this was most common among API (14.2%; p < 0.05) fatal CAN victims. A larger proportion of fatal CAN among API (11.5%) and White (6.7%) victims was believed to be the direct result of the perpetrator’s mental health problems than Black victims (4.0%; p < 0.05). See Table 1 for full results.

### 3.2. Community poverty

From 2003 to 2022, 50.5% of the AI/AN population in NVDRS geographic coverage areas resided in counties with the highest poverty level, followed by Black (40.6%), Hispanic (24.9%), multiracial (23.9%), White (22.3%), and API (15.2%) persons. During this period, 52.7% (n = 58) of AI/AN fatal CAN victims resided in these counties, followed by Black (46.7%; n = 1030), Hispanic (31.3%; n = 266), multiracial (30.9%; n = 82), White (28.7%; n = 711), and API (12.4%; n = 14) victims (Table 2). During this same period, 47.8% (n = 54) of API fatal CAN victims resided in counties with the least poverty, followed by White (17.3%; n = 429), Hispanic (15.3%; n = 130), multiracial (16.6%; n = 44), and Black (10.1%; n = 222) victims. See Supplementary Table 3 for results across all four quartiles.

## 4. Discussion

In this study, during 2003–2022, more than one in three fatal CAN victims resided in communities classified as the most impoverished, disproportionately AI/AN and Black victims. Research shows nonfatal and fatal CAN among children is not independent of broader social and structural factors (e.g., poverty) that impact parenting (Drake et al., 2022; Kim & Drake, 2023; Maguire-Jack & Font, 2017; ***USDHHS, ACF, 2023/2024),*** serving as mechanisms through which CAN has been found to occur (Chandler, Austin, & Shanahan, 2022; Drake et al., 2022; ***USDHHS, ACF, 2023/2024)***. The inequities of residing in communities with the highest poverty level persisted over the entire study period for both AI/AN and Black children, a finding consistent with national child poverty data that show AI/AN, Black, and Hispanic children face far greater exposure to poverty than API and White children (Annie E. Casey Foundation, 2024). In 2022, Black children accounted for the highest proportion of children living in poverty, followed by AI/AN (Annie E. Casey Foundation, 2024), reflective of a history of systemic racism with longstanding and ongoing effects on policies and practices that disadvantage families of color (Williams & Collins, 2001). Economic inequities exacerbate broader patterns of community poverty and negative outcomes associated with concentrated disadvantage (Kneebone & Holmes, 2016; Williams & Collins, 2001), challenging the foundation of strong families and communities (***USDHHS, ACF, 2023/2024)***.

Community poverty, which reflects concentrated disadvantage (e.g., elevated levels of community violence, material hardship, and neighborhood divestment) (Molnar, Buka, Brennan, Holton, & Earls, 2003; U. S. Department of Agriculture, Economic Research Service, 2023), creates pathways through which CAN has been found to occur (Drake et al., 2022; Kim & Drake, 2023; Maguire-Jack & Font, 2017; Molnar et al., 2003). Studies show policies that strengthen economic supports for families often yield significant benefits for children, their families, and communities (Austin et al., 2023; Kovski et al., 2022), underscoring the importance of supporting parents and caregivers by employing antipoverty policies (Kim & Drake, 2019), investing in communities to improve childhood outcomes, and providing equitable access to economic supports and well-resourced communities (Jutte, Badruzzaman, & Thomas-Squance, 2021). In the present study, the largest proportion of fatal CAN victims were aged 1–5 years; a larger proportion of AI/AN and Black children were in this age group; two racial groups that had roughly one in two fatal CAN victims residing in communities with the highest poverty level. Children aged 1–5 years are likely not yet attending grade school, and parents and caregivers living in poverty are least likely to be able to afford quality childcare, highlighting the importance of providing families with access to resources (e.g., early childhood education and high-quality and affordable child care) that have been found to mitigate risk of CAN (Ha, Collins, & Martino, 2015; Johnson-Staub, 2017; Klein, 2011). Prior research found that communities characterized by concentrated poverty and inadequate resources for childcare experienced higher rates of CAN referrals and substantiations, whereas communities with more wealth experienced lower rates (Ha et al., 2015), suggesting that access to childcare services and resources at the community-level are important points of CAN prevention for families with young children and who are residing in communities with high levels of poverty. Providing eligible (e.g., income-driven) families with access to early childhood education (e.g., Early Head Start) has been linked to a wide range of positive child, parent, and family outcomes such as reduced child aggression, parental stress, and parental use of physical punishment (Vogel, Brooks-Gunn, Martin, & Klute, 2013), factors cited as contributors to CAN (Fortson, Klevens, Merrick, Gilbert, & Alexander, 2016; Wilson et al., 2023a). Racial inequities in access to early childhood education and childcare resources exist (Johnson-Staub, 2017), and only 11% of eligible children have access to Early Head Start (National Head Start Association [NHSA], 2024), leaving countless children and families without access to resources that protect against CAN (Johnson-Staub, 2017; NHSA, 2024).

Multiple factors can contribute to risk of fatal CAN victimization (Kennedy, Lazoritz, & Palusci, 2020; Vogel et al., 2013), and the pattern of injuries inflicted upon child victims can take on many forms (Wilson et al., 2023b). As seen in other studies (Wilson et al., 2023b), in the current analysis, the largest proportion of homicides of CAN victims were killed by the perpetrator’s use of personal weapons (i.e., hands, fists, feet), followed by blunt instruments, and firearms. Prior studies suggest the variability in methods by which a perpetrator uses to kill a child in fatal CAN is influenced by factors such as the relationship of the perpetrator to the victim and circumstances or contextual factors (e.g., IPV) surrounding the incident (Daly & Wilson, 1994; Debowska, Boduszek, & Dhingra, 2015; U.S. Department of Health & Human Services, 2024a). Fathers accounted for the largest single category of perpetrators of fatal CAN in the current study. API, multiracial, and White children were significantly more likely to be killed by their fathers than Black children killed by theirs, whereas Black children were significantly more likely to be killed by their mothers than White children being killed by theirs. Research suggests that the characteristics of child homicides committed by mothers are different from those committed by fathers and other male perpetrators (Flynn, Shaw, & Abel, 2013; Porter & Gavin, 2010; Resnick, 1969; Wilson et al., 2023c). As such, studies report mental illness, removal of an unwanted child, CAN, or perceived altruism as some common precipitators in maternal homicides (Debowska et al., 2015; Porter & Gavin, 2010; Resnick, 1969), whereas child homicides committed by fathers often involve IPV, relationship conflict, firearms, and frequently co-occur with perpetrator’s suicide (Debowska et al., 2015; Flynn et al., 2013; Logan, Walsh, Patel, & Hall, 2013; Wilson et al., 2023c). Further findings from prior studies show that almost two-thirds of perpetrators in homicide–suicide incidents with child victims were experiencing intimate partner problems before killing the child (Logan et al., 2013). In the present analysis, larger proportions of API, Hispanic, and White fatal CAN involved IPV, family relationship problems, and involved the use of firearms than Black victims. Taken together, this might help explain why larger proportions of these children were killed by their fathers than Black children killed by theirs. In the present analysis, lower percentages of API (12.4%), Hispanic (31.3%), and White (28.7%) fatal CAN victims resided in communities with higher levels of poverty than Black children (46.7%), which might suggest, for families not experiencing poverty, a convergence of other stressors such as family and intimate partner conflict might be more common in fatal CAN among API, Hispanic, and White children. Teaching safe and healthy relationship skills and conflict resolution across the lifespan, screening for child safety issues among parents at an outpatient pediatric clinic, including exposure to IPV, and supporting IPV survivors to increase safety and lessen harms, have all been proposed as strategies to reduce IPV across the lifespan, including those that involve child victims (Niolon et al., 2017; Thackeray, Hibbard, Dowd, & Committee on Child Abuse and Neglect; Committee on Injury, Violence, and Poison Prevention, 2010).

Further, prior research has documented perpetrator mental illness as a correlate of fatal CAN (Flynn et al., 2013, 2016). Findings from the present study indicate at least 5.6% of the attacks on fatal CAN victims were believed to be the direct result of the perpetrator’s mental illness. Mental illness and crisis episodes often co-occur, especially among suicide decedents (National Alliance on Mental Illness [NAMI], 2024). We found larger proportions of API and White fatal CAN were related to perpetrator’s mental illness or co-occurred with perpetrator’s suicide than Black victims; nearly one in two API fatal CAN victims resided in communities classified as the least impoverished; 17.3% of White and one out of ten Black fatal CAN victims resided in these communities. One study found almost 2 out of 3 of the perpetrators (predominately White males) who committed homicide of a spouse and/or their children before dying by suicide had mental health problems; most of these incidents were precipitated by relationship dissolution (Flynn, Shaw, & Abel, 2007). Prior studies show that not all perpetrators of fatal CAN are receiving mental health treatment at the time of the fatal event (Flynn et al., 2007). In one study that examined 112 perpetrators convicted of infanticide in England and Wales found that 24% had symptoms of mental illness at the time of the offense, 34% had a lifetime history of mental illness, and 14% had been treated for their mental illness (Flynn et al., 2007), highlighting the fact that some perpetrators of fatal CAN may not come to the attention of mental health providers prior to the fatal event. Others (e.g., API communities) might experience barriers (e. g., language barriers, racism, stigma [cultural and societal]) to mental health care (NAMI, 2023). Connecting families who are facing mental health problems or are in crisis and who might not be receiving mental health treatment with emotional and behavioral health supports might aid in preventing fatal and nonfatal CAN.

## 5. Limitations

Findings from this study are subject to a few limitations. First, NVDRS data abstractors are limited to information included in investigative reports, which might not include all characteristics and circumstances for all decedents. Second, due to small counts for AI/AN, API, and multiracial fatal CAN victims, data could not be reported for all years for these groups. Third, this study examined county-level poverty and not children living in poverty; there may have been instances where child victim’s community or household socioeconomic status was different from county-level poverty. Future research might consider a similar analysis using Census Tract-level data to provide a more proximal account of victims’ community-level economic characteristics. Finally, states/jurisdictions joined NVDRS during different years; thus, data were not available for all states/jurisdictions for all data years. Despite these limitations, findings from this study add to the existing literature on the intersection of community poverty and fatal CAN among different racial and ethnic groups.

## 6. Conclusions

In this study, we found the racial and ethnic differences in fatal CAN varied across multiple characteristics and circumstances. Our findings highlight the importance of a comprehensive approach to preventing CAN, such as providing families access to parent training, early education, high-quality and affordable childcare, mental health care, and economic supports, and creating safe, stable, and nurturing relationships and environments for all children (Fortson et al., 2016; Jutte et al., 2021). Further, consistent with other studies, we found that IPV threatens the safety and well-being of children, highlighting the importance of assessing risk of lethal danger of both adult victims and their children in IPV situations (Wilson et al., 2023c).

Additionally, we found racial and ethnic inequities in fatal CAN victims residing in counties classified as the most and least impoverished. Decades of research has documented an association between community poverty and CAN (Coulton, Korbin, Su, & Chow, 1995; Drake et al., 2022; Farrell et al., 2017; Maguire-Jack & Font, 2017). CAN prevention efforts have historically focused on changing individual parent or caregiver behavior, and to a lesser degree, focused on social and structural inequities (e.g., poverty) that amplify families of colors’ need for social services (Child Welfare Information Gateway, 2021; Child Welfare Information Gateway, 2023; USDHHS***, ACF, 2023/2024***). The complex nature of CAN suggests that to prevent CAN, an approach that extends beyond the child welfare system and includes antipoverty policies and programs, antiracism actions, and a broader range of family-serving systems, resources, and supports (e.g., food, housing, economic supports, mental healthcare services, childcare, etc.) for families facing challenges (e.g., mental health problems) and challenging conditions (e.g., poverty) are important to reduce risk for fatal CAN and racial and ethnic inequities in risk (Child Welfare Information Gateway, 2023; Jones, 2021; USDHHS***, ACF, 2023/2024***). Fatal and nonfatal CAN are preventable. Employing multiple strategies, at various levels (e.g., individual, familial, community), might aid in preventing nonfatal and fatal CAN.

## Supplementary Material

Supplementary Table

Supplementary data to this article can be found online at https://doi.org/10.1016/j.chipro.2025.100108.

## Figures and Tables

**Table 1:** Number^a^ and percentage^b^ of fatal abuse and neglect of children aged 0–17 years, by child’s race and ethnicity and child and perpetrator demographic characteristics, weapon type used in fatal event, location of injury, precipitating circumstances,^c^ and incident characteristics, National Violent Death Reporting System, United States,^d^ 2003 to 2022.

	Racial and ethnic groups						
		
Characteristic	American Indian or Alaska Native, non-Hispanic (n=119)	Asian or Pacific Islander, non-Hispanic (n=113)	Black, non-Hispanic (n=2246)	Hispanic^e^ (n=894)	White, non-Hispanic (n=2541)	Multiracial^f^, non-Hispanic (n=269)	Total (n=6182)
							
	No. (%)	No. (%)	No. (%)	No. (%)	No. (%)	No. (%)	No. (%)
Child’s Age (Years)							
<1 year (Infant)	35 (29.4)	29 (25.7)	800 (35.6)	330 (36.9)	904 (35.6)	109 (40.5)	2207 (35.7)
1–5 years^g^	63 (52.9)	37 (32.7)^h,i^	1079 (48.0)^j^	388 (43.4)	1036 (40.8)	112 (41.6)	2715 (43.9)
6–10 years^g^	14 (11.8)	24 (21.2)^h^	206 (9.2)^j^	106 (11.9)	317 (12.5)	25 (9.3)^k^	692 (11.2)
11–15 years^g^	4 (3.4)	19 (16.8)^h,j^	121 (5.4)^j^	58 (6.5)^k^	212 (8.3)	16 (5.9)^k^	430 (7.0)
16–17 years	3 (2.5)	3 (3.5)	40 (1.8)	12 (1.3)	72 (2.8)	7 (2.6)	138 (2.2)
Child’s sex							
Boys	69 (58.0)	61 (54.0)	1301 (57.9)	495 (55.4)	1457 (57.4)	156 (58.0)	3539 (57.3)
Girls	50 (42.0)	52 (46.0)	945 (42.1)	399 (44.6)	1082 (42.6)	113 (42.0)	2641 (42.7)
Weapon type							
Firearm^g^	11 (9.2)	32 (28.3)^h,i^	251 (11.2)^j^	118 (13.2)^j,k^	485 (19.1)	40 (14.9)^k^	937 (15.2)
Personal weapons (e.g., hands, fists, or feet)^g^	40 (33.6)^j^	8 (7.1)	547 (24.4)^j^	228 (25.5)^j^	538 (21.2)	65 (24.2)	1426 (23.1)
Blunt instrument^g^	13 (10.9)	13 (11.5)	469 (20.9)^j^	136 (15.2)^h^	362 (14.2)	49 (18.2)	1042 (16.9)
Sharp instrument^g^	1 (0.8)	14 (12.4)^h,j^	84 (3.7)	39 (4.4)^k^	84 (3.3)	12 (4.5)	234 (3.8)
Hanging, strangulation, or suffocation	14 (11.8)	16 (14.2)^h^	141 (6.3)^j^	78 (8.7)	238 (9.4)	16 (5.9)	503 (8.1)
Shaking (e.g., shaken baby syndrome)	9 (7.6)	7 (6.2)	176 (7.8)	92 (10.3)	243 (9.6)	27 (10.0)	554 (9.0)
Poisoning	2 (1.7)	1 (0.9)	120 (5.3)	35 (3.9)	148 (5.8)	7 (2.6)	313 (5.1)
Intentional neglect (e.g., starving a baby or oneself)^g^	12 (10.1)^j^	3 (2.7)	117 (5.2)	37 (4.1)	109 (4.3)	14 (5.2)	292 (4.7)
Fire, burns, or drowning	5 (4.2)	7 (6.2)	115 (5.1)	38 (4.3)	109 (4.3)	17 (6.3)	291 (4.7)
Other	5 (4.2)	8 (7.1)	71 (3.2)	28 (3.1)	79 (3.1)	3 (1.1)	194 (3.1)
Unknown^l^	7 (5.9)	4 (3.5)	155 (6.9)	65 (7.3)	146 (5.7)	19 (7.1)	396 (6.4)
Location of Injury							
House or apartment	104 (87.4)	100 (88.5)	1912 (85.1)	779 (87.1)	2245 (88.4)	240 (89.2)	5380 (87.0)
Street, road, sidewalk, alley, highway, or freeway	0 (0.0)	1 (0.9)	17 (0.8)	14 (1.6)^j^	14 (0.6)	1 (0.4)	47 (0.8)
Motor vehicle and public transportation or station (e.g., bus, train, plane, airport, depot, taxi)	1 (0.8)	3 (2.7)	71 (3.2)	22 (2.5)	53 (2.1)	7 (2.6)	157 (2.5)
Parking lot, public garage, public transport, or natural area	5 (4.2)	3 (2.7)	51 (2.3)	13 (1.5)	45 (1.8)	4 (1.5)	121 (2.0)
Commercial, retail area, hotel, or motel	1 (0.8)	0 (0.0)	61 (2.7)	16 (1.8)	46 (1.8)	7 (2.6)	131 (2.1)
School	1 (0.8)	0 (0.0)	7 (0.2)	2 (0.2)	17 (0.7)	1 (0.4)	26 (0.4)
Other location	2 (1.7)	3 (2.7)	29 (1.3)	12 (1.3)	43 (1.7)	2 (0.7)	91 (1.5)
Unknown^l^	5 (4.2)	3 (2.7)	100 (4.5)	36 (4.0)	78 (3.1)	7 (2.6)	229 (3.7)
Perpetrator^1^							
Child’s biological father^g^	28 (26.9)^j^	50 (47.2)^h^	616 (31.6)^j^	319 (38.9)^h^	961 (41.7)	106 (43.3)^h^	2080 (37.6)
Child’s biological mother^g^	30 (28.8)	37 (34.9)	548 (28.1)^j^	230 (28.1)	565 (24.5)	56 (22.9)	1466 (26.5)
Mother’s male companion (i.e., boyfriend, stepfather)	20 (19.2)	9 (8.5)	427 (21.9)	151 (18.4)	441 (19.1)	47 (19.2)	1095 (19.8)
Acquaintance or friend	0 (0.0)	1 (0.9)	19 (1.0)	3 (0.4)	8 (0.4)	0 (0.0)	31 (0.6)
Other family member (e.g., uncle)^g^	15 (14.4)^h,j^	4 (3.8)	119 (6.1)	40 (4.9)^i^	121 (5.2)	13 (5.3)^i^	312 (5.6)
Babysitter	3 (2.9)	2 (1.9)	84 (4.3)	38 (4.6)	103 (4.5)	11 (4.5)	241 (4.4)
Other person known to victim	2 (1.9)	1 (0.9)	42 (2.2)	9 (1.1)	29 (1.3)	1 (0.4)	84 (1.5)
Unknown^l^	6 (5.8)	2 (1.9)	94 (4.8)	29 (3.5)	78 (3.4)	11 (4.5)	220 (4.0)
Perpetrator Sex^m^							
Male^g^	65 (54.6)	62 (54.9)	1240 (55.2)^j^	530 (59.3)	1590 (62.6)	176 (65.4)	3663 (59.3)
Female^g^	41 (34.4)	45 (39.8)	724 (32.2)^j^	286 (32.0)	753 (29.6)	73 (27.1)	1922 (31.1)
Perpetrator Age (years)							
<18 years^g^	4 (3.4)	0 (0.0)	52 (2.3)	30 (3.4)^j^	37 (1.5)	7 (2.6)	130 (2.1)
18–24 years^g^	19 (16.0)	13 (11.5)^h^	517 (23.0)^j^	204 (22.8)^j,k^	487 (19.2)	54 (20.1)	1294 (20.9)
25–44 years	43 (36.1)	55 (48.7)	852 (37.9)	361 (40.4)	1174 (46.2)	132 (49.1)	2617 (42.3)
≥45 years^g^	7 (5.9)	20 (17.7)^h,j^	91 (4.1)^j^	40 (4.5)^j,k^	234 (9.2)	11 (4.1)^j,k^	403 (6.5)
Unknown^l^	46 (38.7)	25 (22.1)	734 (32.7)	259 (29.0)	609 (24.0)	65 (24.2)	1738 (28.1)
Interpersonal							
Intimate partner violence-related^g^	10 (9.2)	35 (33.0)^h,i,j^	225 (10.8)^j^	137 (16.4)^h,k^	449 (19.1)	40 (15.8)^k^	896 (15.6)
Family relationship problem^g,n^	7 (8.5)	14 (16.5)^h^	112 (7.2)^j^	88 (15.5)^h,j^	182 (10.5)	13 (7.2)	416 (9.9)
Victim of interpersonal violence during past month^n^	11 (13.4)	9 (10.6)	133 (8.5)	60 (10.6)	186 (10.7)	14 (7.8)	413 (9.8)
Life stressor							
Argument or conflict	15 (13.8)	22 (20.8)	424 (20.3)	205 (24.6)	502 (21.4)	60 (23.7)	1228 (21.4)
Crisis during previous or upcoming 2 weeks^g^	3 (2.8)	15 (14.2)^h,j^	69 (3.3)	56 (6.7)^h^	106 (4.5)	12 (4.7)^k^	261 (4.5)
Homicide event							
Injury occurred at the victim’s residence^g^	95 (79.8)	89 (78.8)	1584 (70.5)^j^	686 (76.7)^h^	1955 (76.9)	212 (78.8)	4621 (74.7)
Homicide/suicide incident^g,o^	8 (6.7)	43 (38.1)^h,j^	131 (5.8)^j^	124 (13.9)^h,j,k^	511 (20.1)	36 (13.4)^h,k^	853 (13.8)
Mentally ill suspect^g,p^	3 (2.5)	13 (11.5)^h^	89 (4.0)^j^	52 (5.8)	169 (6.7)	19 (7.1)	345 (5.6)
Abuse and neglect-related							
History of child abuse or neglect^q^	16 (23.9)	10 (13.5)	275 (20.7)	100 (20.7)	287 (20.1)	17 (12.6)	705 (20.1)
**Total**	**119 (1.9)**	**113 (1.8)**	**2246 (36.3)**	**894 (14.5)**	**2541 (41.1)**	**269 (4.4)**	**6182 (100)**

a Excludes n = 42 child decedents with missing, unknown, other, or unspecified race and ethnicity. A total of 6182 fatal child abuse and neglect cases were included in the pairwise comparison analysis.

b Percentages might not total 100% due to rounding.

c Precipitating circumstances are not mutually exclusive; thus, fatal child abuse and neglect victims may have one or more circumstances.

d Data for this study come from the following states/jurisdictions: Alaska, Maryland, Massachusetts, New Jersey, Oregon, South Carolina, and Virginia (2003–2022); Colorado, Georgia, North Carolina, Oklahoma, Rhode Island, and Wisconsin (2004–2022); Kentucky, New Mexico, and Utah (2005–2022); Ohio (2011–2022), Michigan (2014–2022); New York (2015–2018; 2020–2022); Hawaii (2015, 2016, 2019, 2022); Arizona, Connecticut, Kansas, Maine, Minnesota, New Hampshire, and Vermont (2015–2022); Illinois, Indiana, Iowa, Pennsylvania, and Washington (2016–2022); California, Delaware, District of Columbia, Nevada, Puerto Rico, and West Virginia (2017–2022); Alabama, Louisiana, Missouri, and Nebraska (2018–2022); Montana, North Dakota, and Wyoming (2019–2022); Arkansas, Idaho, Mississippi, South Dakota, Tennessee, and Texas (2020–2022); and Florida (2022).

e Children of Hispanic or Latino ethnicity might be of any race.

f Fatal child abuse and neglect victims with two or more races.

g Characteristic with a statistically significant result.

h Significantly different from non-Hispanic, Black fatal child abuse and neglect victims; p-value of <0.05 was considered statistically significant.

i Significantly different from non-Hispanic, American Indian/Alaska Native fatal child abuse and neglect victims; p-value of <0.05 was considered statistically significant.

j Significantly different from non-Hispanic, White fatal child abuse and neglect victims; p-value of <0.05 was considered statistically significant.

k Significantly different from non-Hispanic, Asian or Pacific Islander fatal child abuse and neglect victims; p-value of <0.05 was considered statistically significant.

l Victim and perpetrator characteristics with missing or unknown data were excluded from the pairwise comparison analysis.

m Perpetrator sex was known in 90.3% (n = 5585) fatal child abuse and neglect cases.

n Data collected for homicides since 2009. Denominator is fatal child abuse and neglect cases during 2009–2022 (n = 4213).

o Homicide/suicide refers to fatal child abuse and neglect cases where the suspect perpetrated the fatal child abuse and neglect and then died by suicide.

p The perpetrator’s attack on the child victim was believed to be the direct result of a mental illness (e.g., schizophrenia or other psychotic condition, depression, or posttraumatic stress disorder).

q Data collected for homicides since 2013. Denominator is fatal child abuse and neglect cases during 2013–2022 (n = 3516).

**Table 2:** Number and percentage^a^ of fatal abuse and neglect of children aged 0–17 years residing in community poverty and surrounding poverty in NVDRS geographic coverage areas^b^ by child’s race and ethnicity, year, and poverty quartile, National Violent Death Reporting System, United States,^c^ 2003 to 2022.

Child’s Race and Ethnicity	Year																			

	2003 to 2007				2008 to 2012				2013 to 2017				2018 to 2022				Overall (2003–2022)			
				
	Quartile 1		Quartile 4		Quartile 1		Quartile 4		Quartile 1		Quartile 4		Quartile 1		Quartile 4		Quartile 1		Quartile 4	
									
	no. (%) of fatal CAN cases	% of Pop. in poverty quartile^d^	no. (%) of fatal CAN cases	% of pop. in poverty quartile^d^	no. (%) of fatal CAN cases	% of pop. in poverty quartile^d^	no. (%) of fatal CAN cases	% of pop. in poverty quartile^d^	no. (%) of fatal CAN cases	% of pop. in poverty quartile^d^	no. (%) of fatal CAN cases	% of pop. in poverty quartile^d^	no. (%) of fatal CAN cases	% of pop. in poverty quartile^d^	no. (%) of fatal CAN cases	% of pop. in poverty quartile^d^	no. (%) of fatal CAN cases	% of pop. in poverty quartile^d^	no. (%) of fatal CAN cases	% of pop. in poverty quartile^d^
**American Indian or Alaska Native, non-Hispanic,**	e	6.9	14 (82.4)	54.8	e	10.4	11 (57.9)	50.2	e	10.6	14 (50.0)	50.4	e	12.2	19 (41.3)	49.2	e	10.7	58 (52.7)	50.5
**Asian Pacific Islander, non-Hispanic**	e	44.0	e	14.2	e	46.4	e	13.4	15 (42.9)	37.8	e	16.5	28 (48.3)	33.6	e	15.1	54 (47.8)	37.1	14 (12.4)	15.2
**Black or African American, non-Hispanic**	31 (10.9)	15.4	116 (40.7)	40.3	38 (11.1)	19.3	157 (45.9)	39.0	62 (10.7)	17.6	279 (48.0)	40.8	91 (9.1)	15.5	478 (47.8)	41.2	222 (10.1)	16.7	1030 (46.7)	40.6
**Hispanic or Latino** ^f^	26 (17.2)	20.8	54 (35.8)	26.2	12 (10.1)	21.8	41 (34.5)	24.1	30 (13.3)	20.2	64 (28.3)	25.8	62 (17.5)	19.0	107 (30.1)	24.5	130 (15.3)	19.8	266 (31.3)	24.9
**White, non-Hispanic**	70 (19.8)	26.4	102 (28.8)	21.7	70 (16.6)	26.1	105 (24.9)	22.1	116 (16.5)	26.4	184 (26.2)	22.0	173 (17.2)	27.9	320 (31.9)	22.7	429 (17.3)	27.0	711 (28.7)	22.3
**Multiracial, non-Hispanic**	e	23.9	11 (33.3)	26.6	11 (18.3)	25.9	20 (33.3)	24.6	e	26.0	10 (22.7)	23.8	27 (21.1)	26.7	41 (32.0)	23.3	44 (16.6)	26.2	82 (30.9)	23.9
**Total**	134 (15.8)	24.6	298 (35.1)	25.1	138 (14.2)	25.2	336 (34.5)	24.8	231 (14.3)	24.9	554 (34.3)	25.0	382 (14.8)	25.1	973 (37.6)	25.2	885 (14.7)	25.0	2161 (35.9)	25.1

**Abbreviations:** CAN (Child Abuse and Neglect), NVDRS (National Violent Death Reporting System); Q (Quartile).

a Total number of fatal CAN victims included in the community poverty analysis is N = 6025 (Q1: n = 885; Q2: n = 1338; Q3: n = 1641; Q4: n = 2161). Percentages might not total 100% due to rounding.

b Surrounding poverty only includes counties participating in NVDRS programs. Quartile 1 includes counties in the lowest poverty level. Quartile 4 includes counties in the highest poverty level.

c Data for this study come from the following states/jurisdictions: Alaska, Maryland, Massachusetts, New Jersey, Oregon, South Carolina, and Virginia (2003–2022); Colorado, Georgia, North Carolina, Oklahoma, Rhode Island, and Wisconsin (2004–2022); Kentucky, New Mexico, and Utah (2005–2022); Ohio (2011–2022), Michigan (2014–2022); New York (2015–2018; 2020–2022); Hawaii (2015, 2016, 2019, 2022); Arizona, Connecticut, Kansas, Maine, Minnesota, New Hampshire, and Vermont (2015–2022); Connecticut (2015–2021); Illinois, Indiana, Iowa, Pennsylvania, and Washington (2016–2022); California, Delaware, District of Columbia, Nevada, and West Virginia (2017–2022); Alabama, Louisiana, Missouri, and Nebraska (2018–2022); Montana, North Dakota, and Wyoming (2019–2022); Arkansas, Idaho, Mississippi, South Dakota, Tennessee, and Texas (2020–2022); and Florida (2022). Poverty and population estimates data to examine community level poverty for Puerto Rico were not available. In 2022, approved by the U.S. Census Bureau, Connecticut adopted nine planning regions as county-equivalent geographic units, replacing data collected at the county-level. This change prohibited us from including Connecticut’s 2022 fatal CAN cases in the community poverty analysis.

d Percent of population in poverty quartiles only includes population residing in counties participating in NVDRS programs.

e Number and percentages are not reported when the number of fatal child abuse and neglect cases is < 10.

f Children of Hispanic or Latino ethnicity might be of any race.

## Abbreviations:

AI/AN: American Indian or Alaska Native
API: Asian or Pacific Islander
CAN: Child Abuse and Neglect
CDC: Centers for Disease Control and Prevention
FIPS: Federal Information Processing System
NVDRS: National Violent Death Reporting System
NH: non-Hispanic
Q: Quartile
U.S.: United States
