# Supplementary material for: Racial and ethnic differences in fatal child abuse and neglect and the intersection of community poverty: U.S., 2003 to 2022
Source: Child Prot Pract. Author manuscript; Available in PMC 2025 Sep 9. (PMC12416292; doi:10.1016/j.chipro.2025.100108)
Supplement: Supplementary Table [file NIHMS2101494-supplement-Supplementary_Table.docx]

**Supplementary Table 1**

**National Violent Death Reporting System (NVDRS) Participation and Geographic Coverage Area for States/Jurisdictions: 2003 to 2022**

| **State/jurisdiction** | **2003** | **2004** | **2005** | **2006** | **2007** | **2008** | **2009** | **2010** | **2011** | **2012** | **2013** | **2014** | **2015** | **2016** | **2017** | **2018** | **2019** | **2020** | **2021** | **2022** |
| --- | --- | --- | --- | --- | --- | --- | --- | --- | --- | --- | --- | --- | --- | --- | --- | --- | --- | --- | --- | --- |
| **Alabama** |  |  |  |  |  |  |  |  |  |  |  |  |  |  |  | X | X | X | X | X |
| **Alaska** | X | X | X | X | X | X | X | X | X | X | X | X | X | X | X | X | X | X | X | X |
| **Arizona** |  |  |  |  |  |  |  |  |  |  |  |  | X | X | X | X | X | X | X | X |
| **Arkansas** |  |  |  |  |  |  |  |  |  |  |  |  |  |  |  |  |  | X | X | X |
| **California** |  |  |  |  |  |  |  |  |  |  |  |  |  |  | X^a^ | X^b^ | X^c^ | X^d^ | X^e^ | X^f^ |
| **Colorado** |  | X | X | X | X | X | X | X | X | X | X | X | X | X | X | X | X | X | X | X |
| **Connecticut** |  |  |  |  |  |  |  |  |  |  |  |  | X | X | X | X | X | X | X | X^g^ |
| **Delaware** |  |  |  |  |  |  |  |  |  |  |  |  |  |  | X | X | X | X | X | X |
| **District of Columbia** |  |  |  |  |  |  |  |  |  |  |  |  |  |  | X | X | X | X | X | X |
| **Florida** |  |  |  |  |  |  |  |  |  |  |  |  |  |  |  |  |  | 0^h^ | 0^h^ | X^i^ |
| **Georgia** |  | X | X | X | X | X | X | X | X | X | X | X | X | X | X | X | X | X | X | X |
| **Hawaii** |  |  |  |  |  |  |  |  |  |  |  |  | X | X | 0^h^ | 0^h^ | X | 0^h^ | 0^h^ | X |
| **Idaho** |  |  |  |  |  |  |  |  |  |  |  |  |  |  |  |  |  | X | X | X |
| **Illinois** |  |  |  |  |  |  |  |  |  |  |  |  |  | X^j^ | X^k^ | X^l^ | X^m^ | X | X | X |
| **Indiana** |  |  |  |  |  |  |  |  |  |  |  |  |  | X | X | X | X | X | X | X |
| **Iowa** |  |  |  |  |  |  |  |  |  |  |  |  |  | X | X | X | X | X | X | X |
| **Kansas** |  |  |  |  |  |  |  |  |  |  |  |  | X | X | X | X | X | X | X | X |
| **Kentucky** |  |  | X | X | X | X | X | X | X | X | X | X | X | X | X | X | X | X | X | X |
| **Louisiana** |  |  |  |  |  |  |  |  |  |  |  |  |  |  |  | X | X | X | X | X |
| **Maine** |  |  |  |  |  |  |  |  |  |  |  |  | X | X | X | X | X | X | X | X |
| **Maryland** | X | X | X | X | X | X | X | X | X | X | X | X | X | X | X | X | X | X | X | X |
| **Massachusetts** | X | X | X | X | X | X | X | X | X | X | X | X | X | X | X | X | X | X | X | X |
| **Michigan** |  |  |  |  |  |  |  |  |  |  |  | X | X | X | X | X | X | X | X | X |
| **Minnesota** |  |  |  |  |  |  |  |  |  |  |  |  | X | X | X | X | X | X | X | X |
| **Mississippi** |  |  |  |  |  |  |  |  |  |  |  |  |  |  |  |  |  | X | X | X |
| **Missouri** |  |  |  |  |  |  |  |  |  |  |  |  |  |  |  | X | X | X | X | X |
| **Montana** |  |  |  |  |  |  |  |  |  |  |  |  |  |  |  |  | X | X | X | X |
| **Nebraska** |  |  |  |  |  |  |  |  |  |  |  |  |  |  |  | X | X | X | X | X |
| **Nevada** |  |  |  |  |  |  |  |  |  |  |  |  |  |  | X | X | X | X | X | X |
| **New Hampshire** |  |  |  |  |  |  |  |  |  |  |  |  | X | X | X | X | X | X | X | X |
| **New Jersey** | X | X | X | X | X | X | X | X | X | X | X | X | X | X | X | X | X | X | X | X |
| **New Mexico** |  |  | X | X | X | X | X | X | X | X | X | X | X | X | X | X | X | X | X | X |
| **New York** |  |  |  |  |  |  |  |  |  |  |  |  | X | X | X | X | 0^h^ | X | X | X |
| **North Carolina** |  | X | X | X | X | X | X | X | X | X | X | X | X | X | X | X | X | X | X | X |

| **State/Jurisdiction** | **2003** | **2004** | **2005** | **2006** | **2007** | **2008** | **2009** | **2010** | **2011** | **2012** | **2013** | **2014** | **2015** | **2016** | **2017** | **2018** | **2019** | **2020** | **2021** | **2022** |
| --- | --- | --- | --- | --- | --- | --- | --- | --- | --- | --- | --- | --- | --- | --- | --- | --- | --- | --- | --- | --- |
| **North Dakota** |  |  |  |  |  |  |  |  |  |  |  |  |  |  |  |  | X | X | X | X |
| **Ohio** |  |  |  |  |  |  |  |  | X | X | X | X | X | X | X | X | X | X | X | X |
| **Oklahoma** |  | X | X | X | X | X | X | X | X | X | X | X | X | X | X | X | X | X | X | X |
| **Oregon** | X | X | X | X | X | X | X | X | X | X | X | X | X | X | X | X | X | X | X | X |
| **Pennsylvania** |  |  |  |  |  |  |  |  |  |  |  |  |  | X^n^ | X^o^ | X^p^ | X^q^ | X | X | X |
| **Puerto Rico** |  |  |  |  |  |  |  |  |  |  |  |  |  |  | X | X | X | X | X | X |
| **Rhode Island** |  | X | X | X | X | X | X | X | X | X | X | X | X | X | X | X | X | X | X | X |
| **South Carolina** | X | X | X | X | X | X | X | X | X | X | X | X | X | X | X | X | X | X | X | X |
| **South Dakota** |  |  |  |  |  |  |  |  |  |  |  |  |  |  |  |  |  | X | X | X |
| **Tennessee** |  |  |  |  |  |  |  |  |  |  |  |  |  |  |  |  |  | X | X | X |
| **Texas** |  |  |  |  |  |  |  |  |  |  |  |  |  |  |  |  |  | X^r^ | X^s^ | X^t^ |
| **Utah** |  |  | X | X | X | X | X | X | X | X | X | X | X | X | X | X | X | X | X | X |
| **Vermont** |  |  |  |  |  |  |  |  |  |  |  |  | X | X | X | X | X | X | X | X |
| **Virginia** | X | X | X | X | X | X | X | X | X | X | X | X | X | X | X | X | X | X | X | X |
| **Washington** |  |  |  |  |  |  |  |  |  |  |  |  |  | X^u^ | X^v^ | X | X | X | X | X |
| **West Virginia** |  |  |  |  |  |  |  |  |  |  |  |  |  |  | X | X | X | X | X | X |
| **Wisconsin** |  | X | X | X | X | X | X | X | X | X | X | X | X | X | X | X | X | X | X | X |
| **Wyoming** |  |  |  |  |  |  |  |  |  |  |  |  |  |  |  |  | X | X | X | X |
| **TOTAL** | **7** | **13** | **16** | **16** | **16** | **16** | **16** | **16** | **17** | **17** | **17** | **18** | **27** | **32** | **37** | **41** | **44** | **50** | **50** | **52** |

**Abbreviations**: NVDRS; National Violent Death Reporting System

**X** without a superscripted footnote denotes statewide data collection, which includes data in all counties within that state; **0**: No data included.

**California**

**^a^2017: Four counties:** (Los Angeles, Riverside, San Francisco, and Santa Clara). These 4 counties represented 30.1% of California’s population.

**^b^2018: Twenty-one counties:** (Amador, Butte, Fresno, Humboldt, Imperial, Kern, Kings, Lake, Los Angeles, Marin, Mono, Placer, Sacramento, San Benito, San Mateo, San Diego, San Francisco, Shasta, Siskiyou, Ventura, and Yolo). These 21 counties represented 54.0% of California’s population.

**^c^2019: Thirty counties:** (Amador, Butte, Colusa, Fresno, Glenn, Humboldt, Imperial, Kern, Kings, Lassen, Lake, Los Angeles, Marin, Modoc, Mono, Orange, Placer, Sacramento, San Benito, San Francisco, San Mateo, Santa Cruz, Shasta, Siskiyou, Solano, Sonoma, Tehama, Trinity, Ventura, and Yolo). These 30 counties represented 57.0% of California’s population.

**^d^2020: Thirty-five counties:** (Amador, Butte, Colusa, Contra Costa, Fresno, Glenn, Humboldt, Imperial, Kern, Kings, Lassen, Lake, Los Angeles, Marin, Mendocino, Merced, Modoc, Mono, Orange, Placer, Sacramento, San Benito, San Diego, San Francisco, San Mateo, Santa Cruz, Shasta, Siskiyou, Solano, Sonoma, Stanislaus, Tehama, Trinity, Ventura, and Yolo). These 35 counties represented 70.6% of California’s population.

**^e^2021: Thirty-one counties:** (Amador, Butte, Colusa, Fresno, Glenn, Humboldt, Imperial, Kings, Lake, Lassen, Los Angeles, Mendocino, Merced, Modoc, Mono, Orange, Placer, Sacramento, San Benito, San Diego, San Francisco, San Luis Obispo, San Mateo, Santa Cruz, Shasta, Siskiyou, Solano, Sonoma, Tehama, Ventura, and Yolo). These 31 counties represented 63.8% of California’s population.

**^f^2022: Thirty-two counties:** (Alpine, Butte, Colusa, Fresno, Glenn, Humboldt, Inyo, Kings, Lake, Lassen, Los Angeles, Mendocino, Merced, Modoc, Mono, Nevada, Orange, Placer, Sacramento, San Benito, San Diego, San Francisco, San Luis Obispo, San Mateo, Santa Clara, Santa Cruz, Shasta, Siskiyou, Solano, Sonoma, Tehama, and Ventura). These 32 counties represented 64.5% of California’s population.

**Connecticut**

**^g^2022:** In 2022, approved by the U.S. Census Bureau, Connecticut adopted nine planning regions as county-equivalent geographic units, replacing data collected at the county-level. This change

prohibited us from including Connecticut’s 2022 fatal CAN cases in the community poverty analysis.

**Florida, Hawaii, and New York**

**^h^**Data for these data years for these states are excluded because the data did not meet the completeness threshold for circumstances in the National Violent Death Reporting System.

**Florida**

**^i^2022: Thirty-two counties:** (Alachua, Baker, Bay, Bradford, Broward, Charlotte, Columbia, DeSoto, Flagler, Gilchrist, Hamilton, Hardee, Highlands, Hillsborough, Indian River, Levy, Manatee, Martin, Miami-Dade, Okeechobee, Orange, Osceola, Palm Beach, Pasco, Pinellas, Polk, Putnam, St. Johns, St. Lucie, Sarasota, Union, and Volusia). These 32 counties represented 66.5% of Florida’s population.

**Illinois**

**^j^2016: Seventeen counties:** (Champaign, Cook, DuPage, Effingham, Kane, Kankakee, Kendall, Lake, McHenry, McLean, Madison, Peoria, St. Clair, Sangamon, Tazewell, Will, and Winnebago). These 17 counties represented 80.9% of Illinois’s population.

**^k^2017: Sixteen counties:** (Cook, DuPage, Effingham, Kane, Kankakee, Kendall, Lake, McHenry, McLean, Madison, Peoria, St. Clair, Sangamon, Tazewell, Will, and Winnebago). These 16 counties represented 79.3% of Illinois’s population.

**^l^2018: Twenty-eight counties:** (Adams, Boone, Champaign, Cook, DuPage, Effingham, Fulton, Kane, Kankakee, Kendall, Lake, Lasalle, Livingston, Logan, McDonough, McHenry, McLean, Macoupin, Madison, Peoria, Perry, Rock Island, St. Clair, Sangamon, Tazewell, Vermillion, Will, and Winnebago). These 28 counties represent 86.0% of Illinois’s population.

**^m^2019: Forty-seven counties:** (Adams, Alexander, Bond, Boone, Brown, Bureau, Champaign, Clay, Cook, DeKalb, Douglas, DuPage, Effingham, Fayette, Fulton, Grundy, Henry, Iroquois, Jackson, Jefferson, Kane, Kankakee, Kendall, Lake, Lasalle, Livingston, Logan, McDonough, McHenry, McLean, Macoupin, Madison, Menard, Peoria, Perry, Piatt, Putnam, Rock Island, St. Clair, Sangamon, Schuyler, Stark, Tazewell, Vermilion, Wayne, Will, and Winnebago). These 47 counties represented 90.0% of Illinois’s population.

**Pennsylvania**

**^n^2016: Twenty-eight counties:** (Adams, Allegheny, Beaver, Berks, Bucks, Cambria, Chester, Cumberland, Dauphin, Delaware, Erie, Fayette, Franklin, Lackawanna, Lancaster, Lawrence, Lebanon, Lehigh, Luzerne, Montgomery, Northampton, Perry, Philadelphia, Schuylkill, Washington, Wayne, Westmoreland, and York). These 28 counties represent 81.6% of Pennsylvania’s population.

**^o^2017: Twenty-eight counties:** (Adams, Allegheny, Beaver, Berks, Bucks, Cambria, Chester, Cumberland, Dauphin, Delaware, Erie, Fayette, Franklin, Lackawanna, Lancaster, Lawrence, Lebanon, Lehigh, Luzerne, Montgomery, Northampton, Perry, Philadelphia, Schuylkill, Washington, Wayne, Westmoreland, and York). These 28 counties represent 81.7% of Pennsylvania’s population.

**^p^2018: Thirty-nine counties:** (Adams, Allegheny, Armstrong, Beaver, Berks, Blair, Bradford, Bucks, Cambria, Carbon, Centre, Chester, Clarion, Clearfield, Clinton, Columbia, Crawford, Dauphin, Delaware, Fayette, Forest, Greene, Indiana, Jefferson, Lackawanna, Lancaster, Lehigh, Luzerne, Monroe, Montgomery, Montour, Northampton, Philadelphia, Schuylkill, Union, Wayne, Westmoreland, Wyoming, and York). These 39 counties represent 82.2% of Pennsylvania’s population.

**^q^2019: Forty counties:** (Adams, Allegheny, Armstrong, Berks, Blair, Bradford, Bucks, Cameron, Cambria, Carbon, Centre, Chester, Clarion, Clearfield, Clinton, Crawford, Dauphin, Delaware, Erie, Fayette, Forest, Greene, Indiana, Jefferson, Lackawanna, Lancaster, Lehigh, Luzerne, Monroe, Montgomery, Northampton, Philadelphia, Schuylkill, Somerset, Sullivan, Susquehanna, Union, Westmoreland, Wyoming, and York). These counties 40 represented 83.0% of Pennsylvania’s population.

**Texas**

**^r^2020: Four counties:** (Bexar, Dallas, Harris, and Tarrant). These four counties represented 39.3% of Texas’s population.

**^s^2021: Thirteen counties:** (Bell, Bexar, Collin, Dallas, Denton, El Paso, Fort Bend, Harris, Montgomery, Nueces, Tarrant, Travis, and Williamson). These 13 counties represented 63.0% of Texas’s population.

**^t^2022: Thirteen counties:** (Bell, Bexar, Collin, Denton, El Paso, Fort Bend, Dallas, Harris, Montgomery, Nueces, Tarrant, Travis, and Williamson). These 13 counties represented 62.3% of Texas’s population.

**Washington**

**^u^2016: Fifteen counties:** (Clallam, Clark, Cowlitz, Grays Harbor, Island, King, Kitsap, Mason, Pierce, Skagit, Snohomish, Spokane, Thurston, Whatcom, and Yakima). These 15 counties represented 86.3% of Washington’s population.

**^v^2017: Twenty-five counties:** (Adams, Benton, Chelan, Clallam, Clark, Cowlitz, Douglas, Franklin, Grant, Grays Harbor, Island, King, Kitsap, Kittitas, Klickitat, Mason, Okanogan, Pierce, Skagit, Snohomish, Spokane, Stevens, Thurston, Whatcom, and Yakima). These 25 counties represented 95.5% of Washington’s population.

**Supplementary Table 2. Poverty quartile ranges, percent of the NVDRS geographic coverage area population in each quartile, and composition of the NVDRS geographic coverage area, by year: NVDRS, United States, 2003 to 2022**

| **Year** | **Poverty quartile ranges for counties in NVDRS geographic coverage areas** | | | | **% of NVDRS geographic coverage area population** | | | | | | | | **Number of counties and population in NVDRS geographic coverage areas** | |
| --- | --- | --- | --- | --- | --- | --- | --- | --- | --- | --- | --- | --- | --- | --- |
|  | **1st quartile** | **2nd quartile** | **3rd quartile** | **4th quartile** | **1st quartile** | | **2nd quartile** | | **3rd quartile** | | **4th quartile** | | **# of Counties** | **Total population** |
|  | **Poverty range** | **Poverty range** | **Poverty range** | **Poverty range** | **Population** | **%** | **Population** | **%** | **Population** | **%** | **Population** | **%** |  |  |
| 2003 | 6.8 or less | 6.9 to 9.3 | 9.4 to 13.1 | 13.2 or greater | 8,978,108 | 24.8 | 9,314,911 | 25.7 | 9,017,675 | 24.9 | 8,922,606 | 24.6 | 301 | 36,233,300 |
| 2004 | 8.0 or less | 8.1 to 10.7 | 10.8 to 14.7 | 14.8 or greater | 16,344,467 | 23.9 | 17,700,967 | 25.8 | 17,314,217 | 25.3 | 17,160,626 | 25.0 | 778 | 68,520,277 |
| 2005 | 7.5 or less | 7.6 to 11.7 | 11.8 to 15.8 | 15.9 or greater | 19,833,594 | 25.5 | 19,827,833 | 25.5 | 18,610,510 | 23.9 | 19,501,740 | 25.1 | 960 | 77,773,677 |
| 2006 | 7.9 or less | 8.0 to 11.7 | 11.8 to 15.6 | 15.7 or greater | 19,195,582 | 24.4 | 20,066,390 | 25.5 | 19,521,647 | 24.8 | 20,028,498 | 25.4 | 960 | 78,812,117 |
| 2007 | 8.0 or less | 8.1 to 11.3 | 11.4 to 15.3 | 15.4 or greater | 19,589,077 | 24.6 | 19,798,585 | 24.8 | 20,356,836 | 25.5 | 20,035,001 | 25.1 | 960 | 79,779,499 |
| 2008 | 8.1 or less | 8.2 to 11.7 | 11.8 to 15.4 | 15.5 or greater | 21,004,279 | 26.0 | 19,489,051 | 24.1 | 20,447,494 | 25.3 | 19,775,459 | 24.5 | 961 | 80,716,283 |
| 2009 | 8.4 or less | 8.5 to 13.2 | 13.3 to 16.9 | 17.0 or greater | 20,331,765 | 24.9 | 20,115,959 | 24.7 | 20,434,696 | 25.0 | 20,704,873 | 25.4 | 962 | 81,587,293 |
| 2010 | 9.6 or less | 9.7 to 14.1 | 14.2 to 18.0 | 18.1 or greater | 20,629,052 | 25.0 | 20,613,558 | 25.0 | 20,946,593 | 25.4 | 20,204,289 | 24.5 | 962 | 82,393,492 |
| 2011 | 10.7 or less | 10.8 to 15.3 | 15.4 to 18.8 | 18.9 or greater | 23,814,983 | 25.2 | 24,100,229 | 25.5 | 23,608,201 | 25.0 | 23,074,703 | 24.4 | 1050 | 94,598,116 |
| 2012 | 10.4 or less | 10.5 to 14.8 | 14.9 to 18.8 | 18.9 or greater | 23,712,431 | 24.9 | 23,463,924 | 24.6 | 23,920,355 | 25.1 | 24,163,785 | 25.4 | 1050 | 95,260,495 |
| 2013 | 10.4 or less | 10.5 to 14.8 | 14.9 to 18.7 | 18.8 or greater | 23,991,991 | 25.0 | 24,217,822 | 25.3 | 23,691,548 | 24.7 | 23,999,631 | 25.0 | 1050 | 95,900,992 |
| 2014 | 10.5 or less | 10.6 to 14.4 | 14.5 to 18.3 | 18.4 or greater | 26,689,876 | 25.1 | 26,469,924 | 24.9 | 27,064,173 | 25.4 | 26,247,236 | 24.7 | 1133 | 106,471,209 |
| 2015 | 9.7 or less | 9.8 to 13.9 | 14.0 to 17.2 | 17.3 or greater | 37,476,712 | 24.9 | 38,519,164 | 25.6 | 36,855,851 | 24.5 | 37,478,439 | 24.9 | 1454 | 150,330,166 |
| 2016 | 9.2 or less | 9.3 to 12.8 | 12.9 to 16.2 | 16.3 or greater | 46,887,332 | 24.9 | 47,296,600 | 25.1 | 46,810,353 | 24.9 | 47,089,911 | 25.0 | 1705 | 188,084,196 |
| 2017 | 9.2 or less | 9.3 to 12.7 | 12.8 to 15.2 | 15.3 or greater | 51,723,320 | 24.7 | 52,594,481 | 25.1 | 52,503,053 | 25.0 | 52,986,674 | 25.3 | 1790 | 209,807,528 |
| 2018 | 9.1 or less | 9.2 to 12.3 | 12.4 to 15.4 | 15.5 or greater | 58,420,582 | 24.8 | 59,596,822 | 25.3 | 58,988,913 | 25.0 | 58,515,425 | 24.8 | 2183 | 235,521,742 |
| 2019 | 8.7 or less | 8.8 to 11.9 | 12.0 to 14.1 | 14.2 or greater | 55,235,451 | 24.8 | 55,492,172 | 24.9 | 55,609,901 | 25.0 | 56,132,047 | 25.2 | 2286 | 222,469,571 |
| 2020 | 8.8 or less | 8.9 to 11.6 | 11.7 to 14.4 | 14.5 or greater | 70,162,345 | 25.1 | 71,959,103 | 25.8 | 68,099,493 | 24.4 | 68,929,527 | 24.7 | 2798 | 279,150,468 |
| 2021 | 9.4 or less | 9.5 to 12.2 | 12.3 to 15.0 | 15.1 or greater | 71,529,701 | 25.2 | 70,994,643 | 25.0 | 69,523,793 | 24.5 | 71,587,717 | 25.2 | 2803 | 283,635,854 |
| 2022 | 9.3 or less | 9.4 to 12.1 | 12.2 to 14.9 | 15.0 or greater | 75,953,882 | 25.4 | 71,587,264 | 23.9 | 74,759,548 | 25.0 | 77,026,655 | 25.7 | 2832 | 299,327,349 |

**Abbreviations: NVDRS: National Violent Death Reporting System**

**Supplementary Table 3. Number and percentage^a^ of fatal abuse and neglect among children aged 0 to 17 years residing in community poverty and surrounding poverty in NVDRS geographic coverage areas^b^ by victim’s race and ethnicity, year, and quartile, NVDRS, United States,^c^ 2003 to 2022**

| **Child’s race and ethnicity** | 2003 | | 2004 | | 2005 | | 2006 | | 2007 | | 2008 | | 2009 | | 2010 | | 2011 | | 2012 | | 2013 | | 2014 | | 2015 | | 2016 | | 2017 | | 2018 | | 2019 | | 2020 | | 2021 | | 2022 | | Total | |
| --- | --- | --- | --- | --- | --- | --- | --- | --- | --- | --- | --- | --- | --- | --- | --- | --- | --- | --- | --- | --- | --- | --- | --- | --- | --- | --- | --- | --- | --- | --- | --- | --- | --- | --- | --- | --- | --- | --- | --- | --- | --- | --- |
|  | No. (%) | % of NVDRS population  ^d^ | No. (%) | % of NVDRS population  ^d^ | No. (%) | % of NVDRS population  ^d^ | No. (%) | % of NVDRS population  ^d^ | No. (%) | % of NVDRS population  ^d^ | No. (%) | % of NVDRS population  ^d^ | No. (%) | % of NVDRS population  ^d^ | No. (%) | % of NVDRS population  ^d^ | No. (%) | % of NVDRS population  ^d^ | No. (%) | % of NVDRS population  ^d^ | No. (%) | % of NVDRS population  ^d^ | No. (%) | % of NVDRS population  ^d^ | No. (%) | % of NVDRS population  ^d^ | No. (%) | % of NVDRS population  ^d^ | No (%) | % of NVDRS population  ^d^ | No. (%) | % of NVDRS population  ^d^ | No. (%) | % of NVDRS population  ^d^ | No. (%) | % of NVDRS population  ^d^ | No. (%) | % of NVDRS population  ^d^ | No. (%) | % of NVDRS population  ^d^ | No. (%) | % of NVDRS population  ^d^ |
| **Asian or Pacific Islander, non-Hispanic** | | | | | | | | | | | | | | | | | | | | | | | | | | | | | | | | | | | | | | | | | | |
| 1st Q | ^e^ | 39.6 | ^e^ | 40.8 | ^e^ | 46.6 | ^e^ | 44.9 | ^e^ | 45.9 | ^e^ | 47.2 | ^e^ | 46.5 | ^e^ | 46.8 | ^e^ | 46.0 | ^e^ | 45.9 | ^e^ | 45.2 | ^e^ | 44.6 | ^e^ | 38.8 | ^e^ | 37.6 | ^e^ | 32.8 | ^e^ | 30.3 | ^e^ | 35.5 | ^e^ | 31.2 | ^e^ | 32.6 | ^e^ | 37.7 | 54 (47.8) | 37.1 |
| 2nd Q | ^e^ | 30.9 | ^e^ | 27.9 | ^e^ | 23.0 | ^e^ | 24.6 | ^e^ | 23.0 | ^e^ | 22.9 | ^e^ | 20.1 | ^e^ | 22.9 | ^e^ | 19.7 | ^e^ | 21.3 | ^e^ | 22.2 | ^e^ | 22.5 | ^e^ | 25.4 | ^e^ | 20.8 | ^e^ | 24.2 | ^e^ | 26.8 | ^e^ | 23.1 | ^e^ | 27.6 | ^e^ | 23.4 | ^e^ | 20.9 | 19 (16.8) | 23.7 |
| 3rd Q | ^e^ | 14.5 | ^e^ | 16.8 | ^e^ | 13.7 | ^e^ | 17.4 | ^e^ | 18.7 | ^e^ | 18.2 | ^e^ | 19.8 | ^e^ | 18.4 | ^e^ | 19.4 | ^e^ | 18.6 | ^e^ | 17.5 | ^e^ | 18.6 | ^e^ | 16.6 | ^e^ | 23.8 | ^e^ | 27.9 | ^e^ | 27.4 | ^e^ | 29.6 | ^e^ | 25.2 | ^e^ | 27.0 | ^e^ | 26.7 | 26 (23.0) | 24.0 |
| 4th Q | ^e^ | 15.1 | ^e^ | 14.5 | ^e^ | 16.7 | ^e^ | 13.0 | ^e^ | 12.4 | ^e^ | 11.7 | ^e^ | 13.6 | ^e^ | 11.9 | ^e^ | 14.9 | ^e^ | 14.3 | ^e^ | 15.1 | ^e^ | 14.3 | ^e^ | 19.2 | ^e^ | 17.8 | ^e^ | 15.1 | ^e^ | 15.5 | ^e^ | 11.7 | ^e^ | 16.1 | ^e^ | 17.0 | ^e^ | 14.8 | 14 (12.4) | 15.2 |
| **American Indian or Alaska Native, non-Hispanic** | | | | | | | | | | | | | | | | | | | | | | | | | | | | | | | | | | | | | | | | | | |
| 1st Q | ^e^ | 7.8 | ^e^ | 7.4 | ^e^ | 5.6 | ^e^ | 5.7 | ^e^ | 8.8 | ^e^ | 9.1 | ^e^ | 9.8 | ^e^ | 9.4 | ^e^ | 11.4 | ^e^ | 12.1 | ^e^ | 12.5 | ^e^ | 12.7 | ^e^ | 8.9 | ^e^ | 10.8 | ^e^ | 9.5 | ^e^ | 10.6 | ^e^ | 10.4 | ^e^ | 14.0 | ^e^ | 12.9 | ^e^ | 12.8 | ^e^ | 10.7 |
| 2nd Q | ^e^ | 29.0 | ^e^ | 18.1 | ^e^ | 18.9 | ^e^ | 16.5 | ^e^ | 15.3 | ^e^ | 15.7 | ^e^ | 16.5 | ^e^ | 15.6 | ^e^ | 19.9 | ^e^ | 13.8 | ^e^ | 14.2 | ^e^ | 15.5 | ^e^ | 17.0 | ^e^ | 14.5 | ^e^ | 17.4 | ^e^ | 19.9 | ^e^ | 17.6 | ^e^ | 19.9 | ^e^ | 20.6 | ^e^ | 21.0 | 20 (18.2) | 17.8 |
| 3rd Q | ^e^ | 21.4 | ^e^ | 22.2 | ^e^ | 20.1 | ^e^ | 21.0 | ^e^ | 18.8 | ^e^ | 23.5 | ^e^ | 22.4 | ^e^ | 26.0 | ^e^ | 22.2 | ^e^ | 21.5 | ^e^ | 22.8 | ^e^ | 22.3 | ^e^ | 24.1 | ^e^ | 25.0 | ^e^ | 21.4 | ^e^ | 19.3 | ^e^ | 18.7 | ^e^ | 18.1 | ^e^ | 19.5 | ^e^ | 18.1 | 26 (23.6) | 21.0 |
| 4th Q | ^e^ | 41.8 | ^e^ | 52.3 | ^e^ | 55.3 | ^e^ | 56.8 | ^e^ | 57.2 | ^e^ | 51.7 | ^e^ | 51.4 | ^e^ | 49.0 | ^e^ | 46.5 | ^e^ | 52.6 | ^e^ | 50.6 | ^e^ | 49.6 | ^e^ | 49.9 | ^e^ | 49.7 | ^e^ | 51.7 | ^e^ | 50.2 | ^e^ | 53.3 | ^e^ | 48.0 | ^e^ | 46.9 | ^e^ | 48.1 | 58 (52.7) | 50.5 |
| **Black, non-Hispanic** | | | | | | | | | | | | | | | | | | | | | | | | | | | | | | | | | | | | | | | | | | |
| 1st Q | ^e^ | 14.6 | ^e^ | 12.6 | ^e^ | 15.3 | ^e^ | 18.1 | ^e^ | 15.8 | ^e^ | 19.9 | ^e^ | 19.4 | ^e^ | 19.7 | ^e^ | 18.9 | ^e^ | 18.6 | ^e^ | 18.2 | ^e^ | 17.9 | 22 (16.8) | 17.9 | 14 (10.3) | 17.5 | 12 (8.6) | 16.8 | 16 (10.0) | 16.9 | 18 (10.3) | 17.3 | 13 (6.9) | 14.0 | 20 (8.0) | 14.6 | 24 (10.6) | 15.6 | 222 (10.1) | 16.7 |
| 2nd Q | ^e^ | 24.2 | ^e^ | 18.8 | ^e^ | 19.4 | 12 (18.2) | 17.9 | 16 (25.4) | 20.8 | 14 (21.5) | 16.7 | ^e^ | 14.4 | ^e^ | 16.0 | ^e^ | 14.0 | ^e^ | 14.7 | 16 (19.3) | 15.0 | 11 (12.1) | 15.3 | 17 (13.0) | 17.1 | 18 (13.2) | 16.3 | 25 (17.9) | 18.4 | 33 (20.6) | 17.0 | 25 (14.4) | 17.1 | 24 (12.8) | 19.7 | 49 (19.6) | 19.2 | 44 (19.4) | 18.1 | 355 (16.1) | 17.6 |
| 3rd Q | ^e^ | 17.7 | 24 (33.8) | 25.3 | 16 (29.6) | 24.9 | 24 (36.4) | 24.9 | 23 (36.5) | 26.5 | 13 (20.0) | 26.0 | 19 (33.3) | 26.0 | 17 (27.9) | 25.0 | 30 (39.5) | 28.9 | 23 (27.7) | 27.0 | 24 (28.9) | 28.4 | 25 (27.5) | 29.0 | 40 (30.5) | 25.0 | 25 (18.4) | 23.4 | 39 (27.9) | 22.0 | 47 (29.4) | 25.9 | 40 (23.0) | 24.4 | 62 (33.0) | 24.6 | 62 (24.8) | 24.0 | 44 (19.4) | 25.7 | 600 (27.2) | 25.2 |
| 4th Q | 16 (51.6) | 43.5 | 35 (49.3) | 43.3 | 24 (44.4) | 40.4 | 21 (31.8) | 39.0 | 20 (31.7) | 36.9 | 31 (47.7) | 37.4 | 22 (38.6) | 40.2 | 29 (47.5) | 39.3 | 29 (38.2) | 38.1 | 46 (55.4) | 39.7 | 37 (44.6) | 38.4 | 47 (51.6) | 37.8 | 52 (39.7) | 40.0 | 79 (58.1) | 42.7 | 64 (45.7) | 42.8 | 64 (40.0) | 40.1 | 91 (52.3) | 41.2 | 89 (47.3) | 41.7 | 119 (47.6) | 42.3 | 115 (50.7) | 40.6 | 1030 (46.7) | 40.6 |
| **Hispanic (any race)^f^** | | | | | | | | | | | | | | | | | | | | | | | | | | | | | | | | | | | | | | | | | | |
| 1st Q | ^e^ | 23.1 | ^e^ | 20.9 | ^e^ | 20.5 | ^e^ | 20.5 | 13 (26.5) | 20.4 | ^e^ | 21.4 | ^e^ | 20.9 | ^e^ | 21.4 | ^e^ | 23.3 | ^e^ | 21.8 | ^e^ | 22.0 | ^e^ | 22.9 | ^e^ | 19.8 | ^e^ | 20.5 | 12 (15.2) | 18.7 | 10 (14.3) | 19.6 | ^e^ | 18.8 | 14 (20.6) | 18.2 | ^e^ | 19.4 | 20 (21.7) | 19.0 | 130 (15.3) | 19.8 |
| 2nd Q | ^e^ | 20.8 | ^e^ | 25.8 | 12 (42.9) | 26.3 | ^e^ | 23.8 | 10 (20.4) | 23.6 | ^e^ | 24.2 | ^e^ | 23.2 | ^e^ | 25.0 | 11 (37.9) | 23.5 | ^e^ | 24.7 | ^e^ | 26.1 | ^e^ | 26.0 | 12 (27.3) | 24.9 | ^e^ | 20.7 | 28 (35.4) | 19.1 | 17 (24.3) | 24.1 | 15 (25.4) | 20.6 | 18 (26.5) | 25.4 | 16 (24.2) | 24.2 | 15 (16.3) | 22.5 | 207 (24.3) | 23.3 |
| 3rd Q | ^e^ | 26.1 | 10 (31.3) | 28.4 | ^e^ | 26.3 | 13 (38.2) | 30.4 | ^e^ | 30.0 | ^e^ | 31.2 | 10 (40.0) | 32.0 | ^e^ | 31.2 | ^e^ | 28.0 | ^e^ | 28.1 | ^e^ | 27.1 | 10 (52.6) | 27.0 | 11 (25.0) | 24.5 | 22 (37.3) | 30.9 | 23 (29.1) | 40.0 | 15 (21.4) | 32.1 | 19 (32.2) | 40.5 | 20 (29.4) | 29.7 | 21 (31.8) | 30.6 | 30 (32.6) | 34.1 | 248 (29.1) | 32.0 |
| 4th Q | ^e^ | 29.9 | 13 (40.6) | 25.0 | ^e^ | 26.8 | ^e^ | 25.3 | 21 (42.9) | 25.9 | ^e^ | 23.2 | ^e^ | 23.8 | ^e^ | 22.4 | 12 (41.4) | 25.2 | 10 (45.5) | 25.4 | ^e^ | 24.9 | ^e^ | 24.1 | 14 (31.8) | 30.8 | 23 (39.0) | 27.9 | 16 (20.3) | 22.2 | 28 (40.0) | 24.2 | 16 (27.1) | 20.1 | 16 (23.5) | 26.7 | 20 (30.3) | 25.9 | 27 (29.3) | 24.4 | 266 (31.3) | 24.9 |
| **White, non-Hispanic** | | | | | | | | | | | | | | | | | | | | | | | | | | | | | | | | | | | | | | | | | | |
| 1st Q | ^e^ | 23.1 | ^e^ | 20.9 | ^e^ | 20.5 | ^e^ | 20.5 | 13 (26.5) | 20.4 | ^e^ | 21.4 | ^e^ | 20.9 | ^e^ | 21.4 | ^e^ | 23.3 | ^e^ | 21.8 | ^e^ | 22.0 | ^e^ | 22.9 | ^e^ | 19.8 | ^e^ | 20.5 | 12 (15.2) | 18.7 | 10 (14.3) | 19.6 | ^e^ | 18.8 | 14 (20.6) | 18.2 | ^e^ | 19.4 | 20 (21.7) | 19.0 | 130 (15.3) | 19.8 |
| 2nd Q | ^e^ | 20.8 | ^e^ | 25.8 | 12 (42.9) | 26.3 | ^e^ | 23.8 | 10 (20.4) | 23.6 | ^e^ | 24.2 | ^e^ | 23.2 | ^e^ | 25.0 | 11 (37.9) | 23.5 | ^e^ | 24.7 | ^e^ | 26.1 | ^e^ | 26.0 | 12 (27.3) | 24.9 | ^e^ | 20.7 | 28 (35.4) | 19.1 | 17 (24.3) | 24.1 | 15 (25.4) | 20.6 | 18 (26.5) | 25.4 | 16 (24.2) | 24.2 | 15 (16.3) | 22.5 | 207 (24.3) | 23.3 |
| 3rd Q | ^e^ | 26.1 | 10 (31.3) | 28.4 | ^e^ | 26.3 | 13 (38.2) | 30.4 | ^e^ | 30.0 | ^e^ | 31.2 | 10 (40.0) | 32.0 | ^e^ | 31.2 | -- | 28.0 | ^e^ | 28.1 | ^e^ | 27.1 | 10 (52.6) | 27.0 | 11 (25.0) | 24.5 | 22 (37.3) | 30.9 | 23 (29.1) | 40.0 | 15 (21.4) | 32.1 | 19 (32.2) | 40.5 | 20 (29.4) | 29.7 | 21 (31.8) | 30.6 | 30 (32.6) | 34.1 | 248 (29.1) | 32.0 |
| 4th Q | ^e^ | 29.9 | 13 (40.6) | 25.0 | ^e^ | 26.8 | ^e^ | 25.3 | 21 (42.9) | 25.9 | ^e^ | 23.2 | ^e^ | 23.8 | ^e^ | 22.4 | 12 (41.4) | 25.2 | 10 (45.5) | 25.4 | ^e^ | 24.9 | ^e^ | 24.1 | 14 (31.8) | 30.8 | 23 (39.0) | 27.9 | 16 (20.3) | 22.2 | 28 (40.0) | 24.2 | 16 (27.1) | 20.1 | 16 (23.5) | 26.7 | 20 (30.3) | 25.9 | 27 (29.3) | 24.4 | 266 (31.3) | 24.9 |
| **Multiracial (two or more races), non-Hispanic** | | | | | | | | | | | | | | | | | | | | | | | | | | | | | | | | | | | | | | | | | | |
| 1st Q | ^e^ | 25.3 | ^e^ | 22.5 | ^e^ | 23.8 | ^e^ | 23.7 | ^e^ | 24.7 | ^e^ | 26.3 | ^e^ | 25.7 | ^e^ | 26.0 | ^e^ | 25.7 | ^e^ | 25.9 | ^e^ | 26.3 | ^e^ | 25.6 | ^e^ | 27.5 | ^e^ | 27.4 | ^e^ | 23.7 | ^e^ | 24.7 | ^e^ | 27.2 | ^e^ | 26.5 | ^e^ | 25.9 | ^e^ | 28.7 | 44 (16.6) | 26.2 |
| 2nd Q | ^e^ | 29.0 | ^e^ | 26.3 | ^e^ | 26.5 | ^e^ | 24.8 | ^e^ | 24.5 | ^e^ | 23.8 | ^e^ | 23.9 | ^e^ | 24.3 | ^e^ | 25.5 | ^e^ | 23.2 | ^e^ | 23.7 | ^e^ | 23.9 | ^e^ | 24.4 | ^e^ | 25.0 | ^e^ | 26.1 | ^e^ | 26.3 | ^e^ | 24.5 | ^e^ | 26.2 | ^e^ | 25.7 | ^e^ | 23.6 | 79 (29.8) | 25.0 |
| 3rd Q | ^e^ | 22.9 | ^e^ | 23.5 | ^e^ | 22.7 | ^e^ | 24.5 | ^e^ | 24.4 | ^e^ | 24.2 | ^e^ | 25.3 | ^e^ | 26.7 | ^e^ | 24.4 | ^e^ | 26.0 | ^e^ | 26.4 | ^e^ | 26.1 | ^e^ | 25.0 | ^e^ | 24.4 | ^e^ | 25.5 | ^e^ | 25.5 | ^e^ | 25.1 | ^e^ | 24.8 | ^e^ | 24.9 | ^e^ | 24.1 | 60 (22.6) | 24.9 |
| 4th Q | ^e^ | 22.8 | ^e^ | 27.7 | ^e^ | 27.1 | ^e^ | 26.9 | ^e^ | 26.3 | ^e^ | 25.7 | ^e^ | 25.1 | ^e^ | 23.0 | ^e^ | 24.4 | ^e^ | 25.0 | ^e^ | 23.5 | ^e^ | 24.4 | ^e^ | 23.1 | ^e^ | 23.2 | ^e^ | 24.7 | ^e^ | 23.5 | ^e^ | 23.2 | 11 (37.9) | 22.5 | ^e^ | 23.5 | 12 (38.7) | 23.6 | 82 (30.9) | 23.9 |

**Abbreviations:** Q; Quartile

^a^Percentages might not total 100% due to rounding.

^b^Surrounding poverty only includes counties participating in NVDRS programs during the study period. Quartile 1 includes counties in the lowest poverty level. Quartile 4 includes counties in the highest poverty level.

^c^Data for this study come from the following states/jurisdictions: Alaska, Maryland, Massachusetts, New Jersey, Oregon, South Carolina, and Virginia (2003–2022); Colorado, Georgia, North Carolina, Oklahoma, Rhode Island, and Wisconsin (2004–2022); Kentucky, New Mexico, and Utah (2005–2022); Ohio (2011–2022), Michigan (2014–2022); New York (2015–2018; 2020–2022); Hawaii (2015, 2016, 2019, 2022); Arizona, Kansas, Maine, Minnesota, New Hampshire, and Vermont (2015–2022); Illinois, Indiana, Iowa, Pennsylvania, and Washington (2016–2022); California, Delaware, District of Columbia, Nevada, and West Virginia (2017–2022); Connecticut (2015–2021); Alabama, Louisiana, Missouri, and Nebraska (2018–2022); Montana, North Dakota, and Wyoming (2019–2022); Arkansas, Idaho, Mississippi, South Dakota, Tennessee, and Texas (2020–2022); and Florida (2022). Poverty and population estimates data to examine community level poverty for Puerto Rico were not available. In 2022, approved by the U.S. Census Bureau, Connecticut adopted nine planning regions as county-equivalent geographic units, replacing data collected at the county-level. This change prohibited us from including Connecticut’s 2022 fatal CAN cases in the community poverty analysis.

^d^Percent of population in poverty quartiles only includes population residing in counties participating in NVDRS programs during the study period.

^e^Number and percentages are not reported when the number of fatal child abuse and neglect cases is <10.

^f^Children of Hispanic or Latino ethnicity might be of any race.
